# Regulated Zn Plating and Stripping by a Multifunctional Polymer‐Alloy Interphase Layer for Stable Zn Metal Anode

**DOI:** 10.1002/advs.202303343

**Published:** 2023-08-13

**Authors:** Junwen Duan, Jiaming Dong, Ruirui Cao, Hao Yang, Kangkang Fang, Ying Liu, Zhitao Shen, Fumin Li, Rong Liu, Huilin Li, Chong Chen

**Affiliations:** ^1^ Henan Key Laboratory of Photovoltaic Materials College of Future Technology Henan University Kaifeng 475000 P. R. China; ^2^ Institute of Solid State Physics Chinese Academy of Sciences Hefei 230031 P. R. China

**Keywords:** aqueous Zn‐ion batteries, polymer‐alloy interphase layers, suppression of dendrites, Zn anode protection

## Abstract

Metallic zinc electrode with a high theoretical capacity of 820 mAh g^−1^ is highly considered as a promising candidate for next‐generation rechargeable batteries. However, the unavoidable hydrogen evolution, uncontrolled dendrite growth, and severe passivation reaction badly hinder its practical implementations. Herein, a robust polymer‐alloy artificial protective layer is designed to realize dendrite‐free Zn metal anode by the integration of zincophilic SnSb nanoparticles with Nafion. In comparison to the bare Zn electrode, the Nafion‐SnSb coated Zn (NFSS@Zn) electrode exhibits lower nucleation energy barrier, more uniform electric field distribution and stronger anti‐corrosion capability, thus availably suppressing the Zn dendrite growth and interfacial side reactions. As a consequence, the NFSS@Zn electrode exhibits a long cycle life over 1500 h at 1 mA cm^−2^ with an ultra‐low voltage hysteresis (25 mV). Meanwhile, when paired with a MnO_2_ cathode, the as‐prepared full cell also demonstrates stable performance for 1000 cycles at 3 A g^−1^. This work provides an inspired approach to boost the performance of Zn anodes.

## Graphical abstract

A polymer‐alloy hybrid artificial layer (Nafion‐SnSb) is deposited on Zn anode through in situ replacement reaction between metal salt solution (SnCl_2_+SbCl_3_) and Zn and subsequent spin‐casting of Nafion layer. The composite protective layer not only effectively reduces the nucleation energy barrier, but also inhibits Zn dendrite formation and interfacial side reactions.

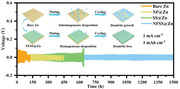

## 1 Introduction

The rapid progress of portable electronics, electric vehicles, and grid‐scale storage devices has imposed a growing demand for high‐energy rechargeable batteries.^[^ ^1^, ^2^, ^3^, ^4^, ^5^, ^6^ ^]^ Zn metal, as the most promising anode material candidate, has gained great attention because of its high operation safety, low cost, low reduction potential (−0.763 V vs the standard hydrogen electrode), and high theoretical specific capacity (5855 mAh cm^−3^, 820 mAh g^−1^).^[^ ^7^, ^8^, ^9^, ^10^, ^11^, ^12^ ^]^ However, the commercial utilization of aqueous Zn‐based batteries (AZBs) suffers from some challenges.^[^ ^13^, ^14^ ^]^ For example, the electrochemical corrosion originated from parasitic reactions between Zn anode and electrolyte will lead to irreversible Zn consumption and obvious capacity decay. In addition, the unavoidable formation of Zn dendrites and “dead Zn” as a result of uneven plating/stripping during repeated cycles also gives rise to internal short circuit and ultimately battery failure.^[^ ^15^, ^16^, ^17^ ^]^

In the past few years, a variety of strategies have been put forward to suppress the dendrite growth and uncontrollable parasitic reactions occurred at the Zn metal anode and electrolytes interface, such as employing 3D currentcollectors,^[^ ^18^, ^19^ ^]^ artificial protective layers,^[^ ^20^, ^21^, ^22^ ^]^ functionalized separators,^[^ ^23^, ^24^, ^25^ ^]^ and electrolyte optimization.^[^ ^26^, ^27^, ^28^ ^]^ Among them, manufacturing of artificial protective layers shows great promise as an effective approach without consuming excess electrolytes.^[^ ^29^, ^30^ ^]^ It enables the Zn metal surface to be passivated and endows the electrode with improved electrochemical performance.^[^ ^31^ ^]^ As an ideal artificial protective layer, it must have the following typical characteristics: i) superior chemical stability to eliminate Zn metal corrosion, ii) high Zn^2+^ conductivity but low electronic conductivity to avoid the “tip effect”, iii) robust mechanical flexibility to tolerate the volume change during continuous cycles and iv) strong affinity to Zn to reduce the interfacial instability.^[^ ^32^, ^33^, ^34^ ^]^ To date, several artificial protective layers like inorganic materials (e.g., graphene,^[^ ^35^ ^]^ ZnO,^[^ ^36^ ^]^ ZrO_2_,^[^ ^37^ ^]^ etc.) and organic polymers (e.g., PEDOT:PSS,^[^ ^38^ ^]^ PPy,^[^ ^39^ ^]^ Nafion,^[^ ^40^ ^]^ etc.) have been widely employed to strengthen the Zn‐electrolyte interface.^[^ ^41^ ^]^ Unfortunately, most of these reported artificial protective layers are hard to satisfy all desirable requirements discussed above.^[^ ^42^ ^]^ The inorganic protective layers generally show good effectiveness in stabilizing Zn anode due to their high ionic conductivity; however, the large volume change of the inorganic protective layer during the electrochemical cycling easily brings about the cracks of the SEI film and reexposes the Zn anode to the electrolyte.^[^ ^43^ ^]^ As for the soft polymer layers with high mechanical strength, they can remarkably alleviate the internal stress at the surface of Zn anode, but tend to exhibit low Zn^2+^ conductivity due to their limited chain mobility.^[^ ^44^, ^45^ ^]^ Recently, the construction of inorganic–organic hybrid artificial layers to combine their respective merits has been proposed and demonstrated to be effective to stabilize the Zn anode and improve the rate capability. Nevertheless, owing to high surface energy of inorganic ceramics and their intrinsically poor compatibility with polymer substances,^[^ ^46^ ^]^ the Zn^2+^ diffusion rate conductivity, flexibility and mechanical properties of current inorganic–organic hybrid artificial layers are yet undesirable.

Herein, a new polymer‐alloy hybrid artificial layer containing SnSb nanoparticles and Nafion (NFSS) is deposited on the surface of Zn metal as robust anode in AZBs. The NFSS layer is facilely formed on the Zn surface through a simple replacement reaction between metal salt solution (SnCl_2_+SbCl_3_) and Zn and subsequent spin‐casting of Nafion layer. The homogeneous and zincophilic SnSb (SS) nanoparticles can provide adequate transportation channels and plentiful Zn^2+^ nucleation sites within Nafion‐SnSb coated Zn (NFSS@Zn) electrode, endowing it with high Zn^2+^ conductivity and low nucleation energy barrier. Meanwhile, the soft Nafion polymers can reinforce the interfacial contact of SS layer with Zn metal anode and selectively shield anions and free H_2_O, enabling the NFSS@Zn electrode to possess superior structural integrity and anti‐corrosion capability, and alleviate the interfacial side reactions. As a result, the NFSS@Zn based symmetric cell achieves uniform Zn plating/stripping at 1 mA cm^−1^ over 1500 h, along with a low overpotential of ≈25 mV. Furthermore, when paired with a MnO_2_ cathode, the NFSS@Zn||MnO_2_ full cell shows higher discharge capacity, longer cycling life, and improved rate performance compared to the bare Zn counterpart.

## 2 Results and Discussion

**Figure 1:**
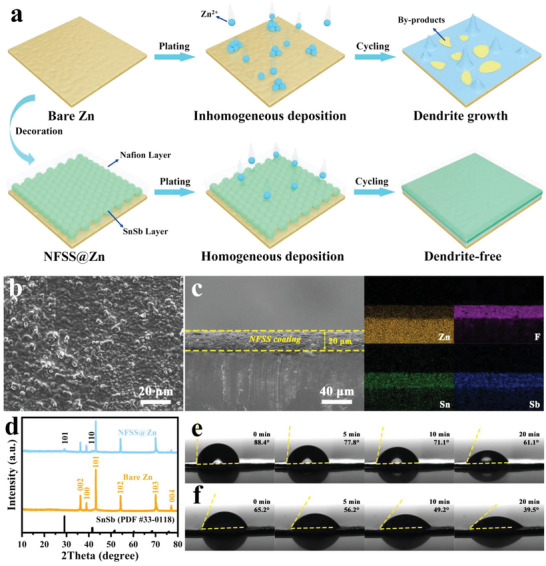
a) Schematic diagram showing Zn deposition using bare Zn and NFSS@Zn electrodes. b) SEM image, c) cross‐sectional SEM image and corresponding elemental mapping of the NFSS@Zn electrode. d) XRD patterns of bare Zn and NFSS@Zn electrodes. In situ contact angle measurement of e) bare Zn and f) NFSS@Zn electrodes.

First, SnSb alloy was deposited onto the surface of Zn foils (SS@Zn) via a simple chemical displacement reaction (5Zn + 2SnCl_2_ + 2SbCl_3_ → 2SnSb + 5ZnCl_2_; φ_Zn2+/Zn_ = −0.763 V, φ_Sn2+_/_Sn_ = −0.14 V, and φ_Sb3+_/_Sb_ = −0.51 V) by simply immersing Zn foil into SnCl_2_‐SbCl_3_‐ethylene glycol solution for 5 min (Figures S1 and S2, Supporting Information).^[^ ^47^ ^]^ Subsequently, the modified Zn foils were treated with Nafion solution and then vacuum dried to obtain NFSS@Zn anodes. The as‐obtained NFSS interfacial layer can not only facilitate homogeneous Zn deposition, but also reduces the direct exposure of Zn anode to electrolytes and effectively inhibit the side reactions during cycling (**Figure** **1**a). SEM images demonstrate that a coating layer consisting of SS and Nafion (NF) with a thickness of ≈20 µm (Figure 1b,c) is formed on the Zn foil surface. Simultaneously, energy‐dispersive X‐ray (EDX) shows the homogeneous distribution of Sn, Sb, and F on the modified Zn foil, which further confirms the uniform formation of NFSS layer on the surface of Zn foil. For better comparison, pristine Nafion coated Zn foil (NF@Zn) was also prepared (See Experimental Section). To gain the phase information, X‐ray diffraction (XRD) patterns for these products were collected in Figure 1d and Figure S3 (Supporting Information). Two peaks at 29.1°and 41.7°, indexed to the (101) and (110) planes of SnSb (JCPDS No. 33–0118), are observed for NFSS@Zn sample. The Zn, Sn, Sb, and F signals from the X‐ray photoelectron spectroscopy (XPS) spectrum of the NFSS@Zn electrode also reveal the successful fabrication of NFSS protective layer on Zn foil (Figure S4, Supporting Information). The wettability of these products was conducted by in situ contact angle tests with 3 m ZnSO_4_ electrolyte. As shown in Figure 1e, the contact angle of the pristine Zn electrode is estimated to be 88.4° in the beginning, while it slowly decreases to 61.1° after 20 min. By contrast, benefiting from the synergistic effects of SnSb alloy and Nafion film (Figure S5, Supporting Information), the initial contact angle of NFSS@Zn one is 65.2° and sharply declines to 39.5° after 20 min, implying its remarkably improved hydrophilicity (Figure 1f). Thus, the improved wettability is helpful to facilitate the infiltration of electrolyte over the surface of NFSS@Zn electrode.

**Figure 2:**
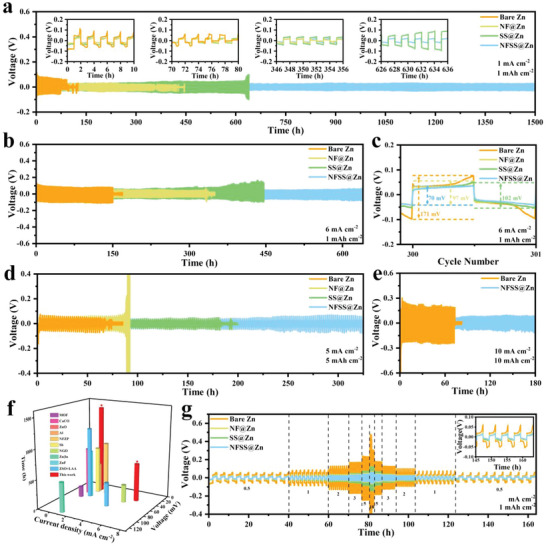
Voltage profiles of bare Zn, NF@Zn, SS@Zn, and NFSS@Zn symmetrical cells at various current densities and plating/stripping capacity, a) 1 mA cm^−2^, 1 mAh cm^−2^; b,c) 6 mA cm^−2^, 1 mAh cm^−2^; d) 5 mA cm^−2^, 5 mAh cm^−2^. e) Polarization voltage of bare Zn and NFSS@Zn symmetrical cells with a current density of 10 mA cm^−2^ for 10 mAh cm^−2^. f) Comparison of the cycling performance of NFSS@Zn electrode with those reported data. g) Rate performance of the symmetrical cells at current densities from 0.5 to 10 mA cm^−2^.

To evaluate the plating/stripping reversibility of bare Zn, NF@Zn, SS@Zn, and NFSS@Zn electrodes, symmetrical coin cells were assembled and cycled at various current densities (**Figure** **2**). The overpotentials of bare Zn, NF@Zn, SS@Zn, and NFSS@Zn electrodes decrease in sequence, indicating few side reactions occurring on NFSS@Zn electrode (Figure 2a). After 75 h, a large voltage fluctuation of bare Zn electrode appears, which is most likely due to the internal short circuit caused by the uncontrolled growth of Zn dendrites. Similar battery failures are observed in NF@Zn and SS@Zn electrodes after 355 and 627 h, respectively. In contrast, the NFSS@Zn electrode delivers a prolonged cycle life (≈1500 h) with a low overpotential (25 mV) under the identical condition. The enhanced cycling stability of NFSS@Zn electrode is further observed at a higher current density of 6 mA cm^−2^ (Figure 2b,c). Moreover, the symmetrical cells of NFSS@Zn electrode were tested at a current density of 5 mA cm^−2^ with a capacity of 5 mAh cm^−2^. As presented in Figure 2d, it can stably operate more than 300 h. Even at 10 mA cm^−2^ for 10 mAh cm^−2^, the NFSS@Zn electrode still runs over 180 h, beyond doubling the cycle lifespan when compared with that of bare Zn (Figure 2e). It is worth noting that the NFSS@Zn electrode exhibits stable cycling performance at an ultrahigh current density of 20 mA cm^−2^ with a limited capacity of 30 mAh cm^−2^ (DOD = 51%) (Figure S6, Supporting Information). These results are far better than most of the recently reported high‐performance Zn anodes (Figure 2f), and fully confirm that the NFSS artificial layer can dramatically boost the cycle stability of Zn electrode.^[^ ^48^, ^49^, ^50^, ^51^, ^52^, ^53^, ^54^, ^55^, ^56^, ^57^, ^58^ ^]^ Besides, significant difference of overpotential can be found in rate performance test (Figure 2g). When the current density is increased from 0.5 to 10 mA cm^−2^, a constant overpotential of 10.8, 15.3, 19.3, 20.7, 22.1, and 44.1 mV can be detected in the NFSS@Zn electrode, much smaller than that of bare Zn, NF@Zn, and SS@Zn electrodes.

**Figure 3:**
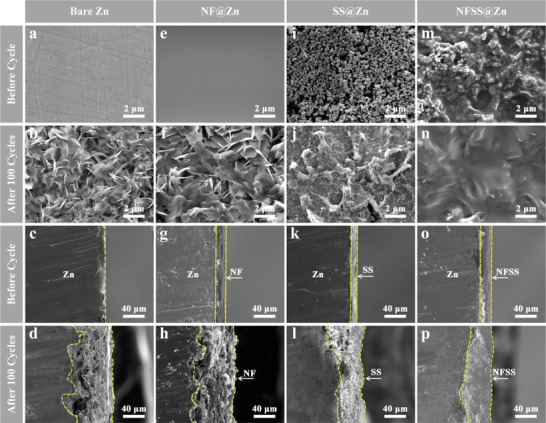
Structure changes of bare Zn, NF@Zn, SS@Zn, and NFSS@Zn before and after 100 cycles in symmetrical cells at a current density of 6 mA cm^−2^. Top‐view and side‐view SEM images of four electrodes, a–d) bare Zn, e–h) NF@Zn, i–l) SS@Zn, and m–p) NFSS@Zn.

To further clarify the mechanism of NFSS coating layer in improving the electrochemical performance of Zn electrode, ex situ SEM and XRD measurements were performed before and after repeated plating/stripping for 100 cycles. As shown in **Figure** **3**a,c, the bare Zn electrode shows a flat and smooth surface without obvious cracks before cycling. After 100 cycles, numerous lamellae in the micrometer level are deposited on the surface of bare Zn electrode, which is ascribed to the side reactions of Zn foil with water and inhomogeneous Zn deposition (Figure 3b,d).^[^ ^59^ ^]^ According to XRD and EDS results (Figures S7 and S8, Supporting Information), these irregular lamellae are assigned to the Zn_4_SO_4_(OH)_6_·5H_2_O, an undesired by‐product of Zn electrode, while the vertical‐growing lamellae are ascribed to Zn dendrites. The by‐product accumulation and dendrites growth lead to the large electrode overpotential and poor cycling life of bare Zn.^[^ ^60^ ^]^ The cross‐section SEM images present the irregular surface after 100 cycles (Figure S9, Supporting Information). After the coating of Nafion, the initial surface seems to be more homogeneous and denser, but there are inadequate nucleation sites for Zn^2+^ (Figure 3e,g). Such a coating layer cannot effectively regulate the Zn plating/striping behavior.^[^ ^61^ ^]^ Thus, it is easier to be punctured by micrometer‐sized protrusions after cycling (Figure 3f,h). For the SS@Zn electrode, the initial surface is found to be covered by a large number of nanoparticles, while there exists abundant pores/voids inside the SS layer (Figure 3i,k). The unique porous structure can allow for electrolyte penetration and provide great possibilities for reacting with Zn foil. As a result, the surface pore/voids are easily occupied by the formation of by‐products upon the repeated plating/stripping cycles, and it has limited effectiveness in inhibiting the side reactions (Figure 3j,l). Notably, the artificial NFSS layer on Zn electrode is highly dense and possesses abundant zincophilic sites, which prevents the permeation of electrolyte and eliminates the side reactions (Figure 3m,o). As expected, the cycled NFSS@Zn electrode still shows a flat surface without distinct protrusion (Figure 3n,p). XRD patterns also prove that the formation of Zn_4_SO_4_(OH)_6_·5H_2_O can be well restrained (Figure S7, Supporting Information). EDS mapping images further show that the Zn deposition on the NFSS@Zn occurs below NF layer with a compact and uniform feature (Figure S10, Supporting Information). The dendrite‐free Zn deposition induced by the NFSS layer guarantees the long‐term and highly reversible Zn plating/stripping process.

**Figure 4:**
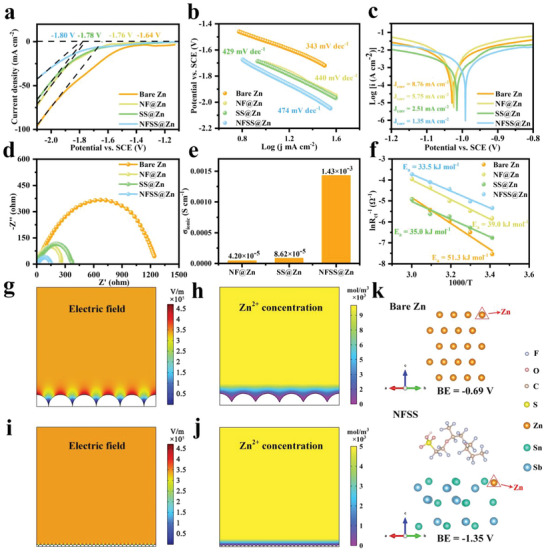
Suppressed electrolyte infiltration and enhanced reaction kinetics in NFSS@Zn. a) Linear sweep voltammetry (LSV), b) Tafel plots, c) linear polarization (LP) curves, d) charge‐transfer resistance (*R*_ct_), e) ionic conductivities (*σ*_ionic_), and f) diffusion energy barriers (*E*_a_). The simulated electric field and Zn^2+^ ions concentration field distribution on g,h) bare Zn and i,j) NFSS@Zn. k) Binding energy of Zn atoms adsorbed on the top sites of bare Zn and NFSS.

To determine the capability of NFSS@Zn electrode in inhibiting the side reactions, linear sweep voltammetry (LSV), linear polarization (LP), and Tafel plots measurements of bare Zn, NF@Zn, SS@Zn, and NFSS@Zn electrodes were performed using three electrode systems. The hydrogen evolution reaction (HER) and Zn plating tend to occur at low potentials in ZnSO_4_, leading to the difficult identification of their onset potentials. 1 m Na_2_SO_4_ was employed as electrolyte in the LSV measurements to migrate the interference of the Zn deposition. Notably, the onset potential of NFSS@Zn electrode is much lower than those of bare Zn, NF@Zn, and SS@Zn electrodes, which indicates the reduced hydrogen evolution activity of NFSS@Zn electrode (**Figure** **4**a). The Tafel slop of those electrodes is found to be in order of NFSS@Zn > SS@Zn > NF@Zn > bare Zn (Figure 4b), which also reflects that the NFSS artificial layer can significantly restrain the hydrogen evolution reaction.^[^ ^21^ ^]^ In addition, we also investigated the Zn corrosion that arises from the oxygen dissolved in the electrolyte by LP curves.^[^ ^31^ ^]^ The NFSS@Zn electrode exhibits a higher corrosion potential of −0.99 V and a lower corrosion current density of 1.35 mA cm^−2^ compared to bare Zn (−1.03 V and 8.76 mA cm^−2^), NF@Zn (−1.02 V and 5.75 mA cm^−2^), and SS@Zn (−1.01 V and 2.51 mA cm^−2^) electrodes, fully indicating the NFSS artificial layer can also strengthen the corrosion resistance (Figure 4c). Meanwhile, the desolvation and transportation kinetics of Zn^2+^ can be accelerated by the introduction of NFSS interfacial layer, as confirmed by electrochemical impedance spectroscopy (EIS) in Figure 4d. The NFSS@Zn electrode presents a smaller charged‐transfer resistance (*R*_ct_) as compared with bare Zn, NF@Zn, and SS@Zn electrodes, showing it owns rapid charge transfer. This is further confirmed by the smallest overpotential in the NFSS@Zn electrode (Figure S11, Supporting Information).^[^ ^62^ ^]^ The electrical conductivity and ionic conductivity (σ_ionic_) of the SS, NF, and NFSS layers were measured and calculated in Figures S12 and S13 (Supporting Information). The value of electrical conductivity of NFSS layer (8.16 × 10^−4^ S cm^−1^) falls between that of NF layer (2.58 × 10^−4^ S cm^−1^) and SS layer (1.01 × 10^−3^ S cm^−1^) (Figure 4d). Nevertheless, compared with those of SS layer (8.62 × 10^−5^ S cm^−1^) and NF layer (4.20 × 10^−5^ S cm^−1^), the σ_ionic_ of NFSS layer (1.43 × 10^−3^ S cm^−1^) is remarkably boosted (Figure 4e). Meanwhile, the activation energy (*E*_a_) was measured by the Arrhenius Equation to gain valuable insight into the effect of the NFSS layer on Zn deposition interface,^[^ ^23^, ^63^ ^]^ as shown in Figure 4f and Figure S14 (Supporting Information). The *E*_a_ for the NFSS@Zn electrode is 33.5 kJ mol^−1^, which is smaller than that for the bare Zn (51.3 kJ mol^−1^), NF@Zn (39 kJ mol^−1^) and SS@Zn (35 kJ mol^−1^) electrodes. Expectedly, such the high ionic conductivity and low charge‐transfer resistance, and low activation energy barrier can promote the homogeneous plating/stripping of Zn in NFSS@Zn during cycling. Furthermore, cyclic voltammetry (CV) curve of the NFSS@Zn electrode exhibits much larger closed area than that of the bare Zn electrode, suggesting more abundant nucleation sites, which favors superior stability of the anode (Figure S15, Supporting Information).^[^ ^32^ ^]^ To analyze the influence of NFSS layer on electric field and Zn^2+^ ions concentration distribution, finite element simulation was conducted. As shown in Figure 4g, Zn^2+^ ions are prone to deposit randomly on the bare Zn surface, leading to a lot of irregular protrusions. These protrusions can increase the field intensity of electrode and promote excessive Zn^2+^ ions to nucleate around the tips with a high activity, which subsequently induces the growth of zinc dendrites. Furthermore, the simulation result of Zn^2+^ ions field distribution reveals that the continuous accumulation of Zn^2+^ ions disrupts the ion distribution,^[^ ^64^ ^]^ ultimately resulting in inhomogeneous Zn deposition (Figure 4h). However, the coating of NFSS layer causes a significant reduction in the surrounding electric field intensity, resulting in a uniform distribution of interfacial electric field (Figure 4i). Benefiting from the homogeneous electric field, Zn^2+^ ions and charges may be uniformly distributed over the NFSS@Zn surface and form dendrite‐free morphology (Figure 4j). The inhibition of Zn dendrite growth by the NFSS artificial layer is further revealed by density functional theory (DFT) in Figure 4k. The binding energy of a Zn atom on the bare Zn surface is calculated to be −0.69 eV (Top), while the binding energy of a Zn atom on the NFSS@Zn surface is −1.35 eV (Top). Such a high‐affinity Zn binding site of NFSS can offer a low energy barrier for Zn deposition, thus driving the adsorption and growth of Zn atoms (Figures S16 and S17, Supporting Information). Based on the results above, constructing a NFSS artificial layer on the surface of Zn foil can suppress the hydrogen evolution and Zn corrosion, and induce the homogenize Zn deposition, which is beneficial for achieving long‐life Zn anode for AZBs.

**Figure 5:**
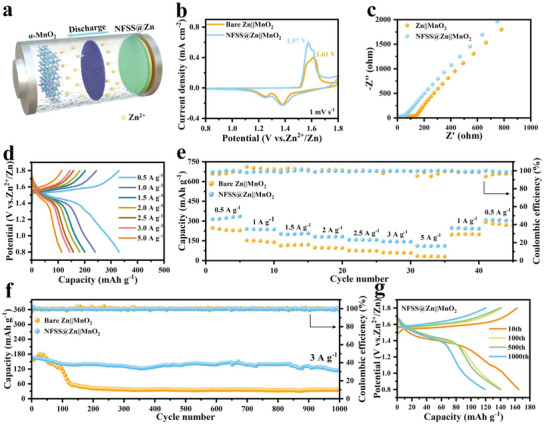
Electrochemical performance of Zn||MnO_2_ batteries with bare Zn and NFSS@Zn anodes. a) Schematic illustration of ZIBs. b) CV curves at 0.1 mV s^−1^. c) Nyquist plots before cycling. d) Corresponding charge/discharge curves at different current densities in the voltage range of 0.8–1.8 V (vs Zn/Zn^2+^). e) Rate performance. f) Long‐term cycling performance at 3 A g^−1^ after 1000 cycles. g) Corresponding charge/discharge curves at different cycles in the voltage range of 0.8–1.8 V (vs Zn/Zn^2+^).

The advantages of NFSS@Zn electrode as anode were further studied by assembling Zn||MnO_2_ full cells, where MnO_2_ nanowire electrode was chosen as cathode (**Figure** **5**a). MnO_2_ was synthesized by a reported hydrothermal method.^[^ ^65^ ^]^ The XRD pattern and SEM images of the MnO_2_ nanowire electrode are displayed in Figures S18 and S19 (Supporting Information). MnSO_4_ (0.1 m) was added to the electrolyte as the additive to inhibit the dissolution of Mn^2+^. Figure 5b shows the cyclic voltammetry profiles of NFSS@Zn||MnO_2_ and Zn||MnO_2_ cells at a scan rate of 0.1 mV s^−1^. Two pairs of characteristic redox peaks are observed in both cells. Obviously, the NFSS@Zn||MnO_2_ cell shows a higher current density as compared to Zn||MnO_2_ cell, revealing an improved electrochemical activity of the NFSS@Zn anode. In addition, a lower voltage polarization is achieved in the NFSS@Zn||MnO_2_ cell, which is consistent with a smaller transfer resistance (Figure 5c). Meanwhile, the NFSS@Zn||MnO_2_ cell delivers average capacities of 313.7, 239.6, 202.2, 182.2 158.2, 145, and 114.2 mAh g^−1^ at 0.5, 1, 1.5, 2, 2.5, 3, and 5 A g^−1^, respectively (Figure 5d). The rate performance of NFSS@Zn||MnO_2_ cell is quite better than that of Zn||MnO_2_ cell (Figure 5e). For NFSS@Zn||MnO_2_ cell, a capacity of 115.7 mAh g^−1^ is maintained after 1000 cycles at a high current density of 3 A g^−1^ (Figure 5f,g), corresponding to a retention rate of 71.8%. In contrast, the capacity of Zn||MnO_2_ cell rapidly decays to 36.2 mAh g^−1^ after 1000 cycles. Furthermore, the SEM images after cycling suggest that the NFSS interfacial layer has a vital role in suppressing side reactions and regulating Zn deposition behaviors (Figures S20–S22, Supporting Information). As a consequence, these results strongly confirm the superiority of NFSS interfacial layer in the terms of stabilizing Zn anode and also implies the applicability of NFSS layer in rechargeable metal‐ion batteries.

## 3 Conclusion

In summary, an NFSS artificial protective layer has been successfully deposited on the surface of Zn meal for achieving highly stable Zn anode. Taking into account the merits of Nafion and zincophilic SnSb, the NFSS layer not only owns massive zincophilic sites, high Zn^2+^ conductivity, and low charge‐transfer resistance, but also exhibits low nucleation energy barrier and uniform electric field distribution. Additionally, the dense NFSS layer can serve as an anti‐corrosion layer to avoid the direct contact of Zn anode with electrolyte, which effectively suppresses the hydrogen evolution reaction and Zn corrosion. Consequently, the NFSS@Zn electrode in the symmetrical cell presents a smaller voltage fluctuation and a longer lifespan of over 1500 h at 1 mA cm^−2^ than the bare Zn electrode. Moreover, the NFSS@Zn||MnO_2_ full cell also delivers an impressive cycling stability with more than 71.8% capacity retention at 3 A g^−1^ after 1000 cycles. This work provides a new route for enhancing the interfacial stability of electrode/electrolyte to achieve high‐performance AZBs.

## 4 Experimental Section

### Preparation of NFSS@Zn Electrode

Zinc foils were washed several times with deionized water and ethanol to eliminate the residual impurities. Then, the treated Zn foils were immersed into 50 mL of 0.5 m antimony chloride (SbCl_3_) and 0.4 m tin (II) chloride (SnCl_2_·H_2_O) ethylene glycol solution at 4 °C for 2 min. Afterward, 40 µL Nafion membrane solution (5%) was spin‐coated on the surface of SS@Zn foil to obtain NFSS@Zn electrode. The SS@Zn electrode was synthesized using the same method without Nafion solution. The NF@Zn electrode was synthesized in the same method without immersion in SbCl_3_‐SnCl_2_ ethylene glycol solution.

### Synthesis of MnO_2_

The MnO_2_ nanowires were fabricated in the same method as previously reported.^[^ ^65^ ^]^ First, 2 mL of 0.5 m hydrochloric acid (HCl) was added to 40 mL of 0.05 m manganese sulfate (MnSO_4_·H_2_O) and then stirred vigorously for 0.5 h at 25 °C. Subsequently, 20 mL of 0.1 m potassium permanganate (KMnO_4_) was added into the solution. The mixed solution was transferred into a Teflon‐lined autoclave. The reaction system was kept at 120 °C for 12 h. Finally, the suspension was collected by centrifugation, washed at least three times with deionized water.

### Characterizations

The morphologies of the Zn foil and modified foils before and after cycling were characterized by scanning electron microscopy (SEM, JEOL JSM‐7001F) equipped with an energy dispersive X‐ray spectrometer. The crystalline structure of the samples was investigated by an X‐ray diffractometer (Bruker D8 Advance) with Cu Kα radiation (λ = 0.15406 nm). The wettability of electrodes was carried out using a contact angle measuring device (Kruss, DSA30). X‐ray photoelectron spectroscopy (XPS) was recorded with an ESCALAB 250Xi (Thermo Fisher) spectrometer.

### Electrochemical Measurements

All the electrochemical performances were measured in CR2032‐type coin cells. The symmetric cells were assembled using bare Zn, NF@Zn, SS@Zn, or NFSS@Zn electrodes as the working and counter electrodes. The galvanostatic charge/discharge (GCD) tests were carried out on a battery tester (LAND CT2001A, China). Cyclic voltammetry (CV), linear sweep voltammetry (LSV), linear polarization (LP) curves, and electrochemical impedance spectroscopy (EIS) were conducted on an electrochemical workstation (CHI 760E, China). Full cells were assembled with NFSS@Zn as the anode, MnO_2_ as the cathode, and 3 m ZnSO_4_ and 0.1 m MnSO_4_ as the electrolyte. The MnO_2_ cathode was prepared by pasting the slurry with 70 wt.% of MnO_2_ nanowires, 20 wt.% of carbon black, and 10 wt.% of polyvinylidene fluoride (PVDF) binder onto a carbon cloth. After that, the coated carbon cloth was dried at 60 °C for 24 h. The cut‐off voltage of NFSS@Zn||MnO_2_ full cell was set to 0.8–1.8 V.

### DFT Calculations

Density functional theory (DFT) calculations were performed using Vienna ab initio Simulation package (VASP).^[^ ^66^ ^]^ In this simulation, the exchange‐correlation function was investigated by employing the generalized gradient approximation (GGA) method with Perdew–Burke–Ernzerhof (PBE) function.^[^ ^67^ ^]^ The projector augmented‐wave method was applied and the cutoff energy of was 450 eV. The dense Monkhorst‐Pack k‐points 2 × 3 × 1 were used for the Brillouin zone. The vacuum slab models with vacuum region of 15 Å above these Zn atoms were used to estimate the adsorption of O atom on bare Zn and NFSS@Zn surfaces. The convergence criterion for energy was set to be 10^−6^ eV, and the residual forces on each atom were reduced below 0.05 eV Å^−1^.

### Electric Field Simulation

A simplified 2D parallel plate capacitor model was employed to analyze the electric field distribution on the Zn anode surface with or without NFSS protective layer. In this model, the length of two electrodes is 5.0 µm and the height is 0.1 µm. The protuberances on bare Zn and NFSS protective layer on NFSS@Zn were represented by semi‐ellipses and semicircles, respectively. The size of protuberances and NFSS were obtained from the SEM results. The voltage hysteresis from symmetric cells (with or without NFSS protective layer) was set as cathodic potential, and a constant of 0 was set as anodic potential.

## Conflict of Interest

The authors declare no conflict of interest.

## Supporting information

Supporting Information

## Data Availability

The data that support the findings of this study are available from the corresponding author upon reasonable request.
