# Supplementary material for: Regulated Zn Plating and Stripping by a Multifunctional Polymer‐Alloy Interphase Layer for Stable Zn Metal Anode
Source: Adv Sci (Weinh). 2023 Aug 13;10(29):2303343. doi: 10.1002/advs.202303343 (PMC10582457; doi:10.1002/advs.202303343)
Supplement: Supplementary file 1 — Supporting Information [file ADVS-10-2303343-s001.pdf]

ADVANCED SCIENCE
Open Access

Supporting Information

for *Adv. Sci.*, DOI 10.1002/advs.202303343

Regulated Zn Plating and Stripping by a Multifunctional Polymer-Alloy Interphase Layer for Stable Zn Metal Anode

Junwen Duan, Jiaming Dong, Ruirui Cao, Hao Yang, Kangkang Fang, Ying Liu, Zhitao Shen*, Fumin Li, Rong Liu, Huilin Li and Chong Chen**

Copyright WILEY-VCH Verlag GmbH & Co. KGaA, 69469 Weinheim, Germany, 2016.

Supporting Information

Regulated Zn plating and stripping by a multifunctional polymer-alloy interphase layer for stable Zn metal anode

Junwen Duan, Jiaming Dong, Ruirui Cao, Hao Yang, Kangkang Fang, Ying Liu, Zhitao Shen*, Fumin Li, Rong Liu, Huilin Li, Chong Chen**

J. W. Duan, J. M. Dong, Dr. R. R. Cao, H. Yang, K. K. Fang, Dr. Y. Liu, Dr. Z. T. Shen, Dr. F. M. Li, Dr. R. Liu, Dr. H. L. Li, Dr. C. Chen

Henan Key Laboratory of Photovoltaic Materials, College of Future Technology, Henan University, Kaifeng 475000, China

E-mail: yliu344@outlook.com; shenzt@vip.henu.edu.cn; chongchen@henu.edu.cn

Dr. C. Chen

Institute of Solid State Physics, Chinese Academy of Sciences, Hefei 230031, PR China

E-mail: chongchen@henu.edu.cn

J. W. Duan and J. M. Dong contributed equally to this work.

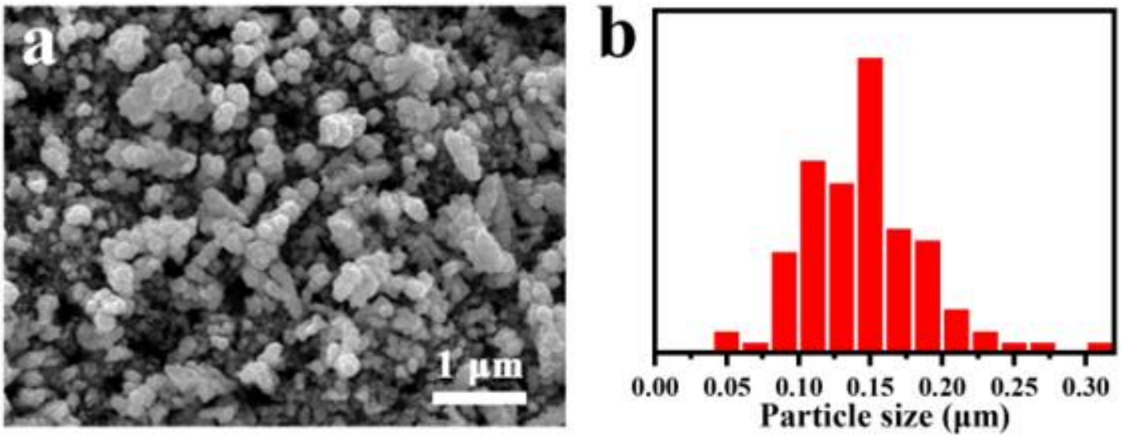

Figure S1. (a) SEM image of SS@Zn electrode. (b) Size distribution of SS nanoparticles on the SS@Zn electrode surface.

As shown in Figure S1, the SS@Zn electrode is covered with a large amount of SS particles with an average size of approximately 0.14 µm.

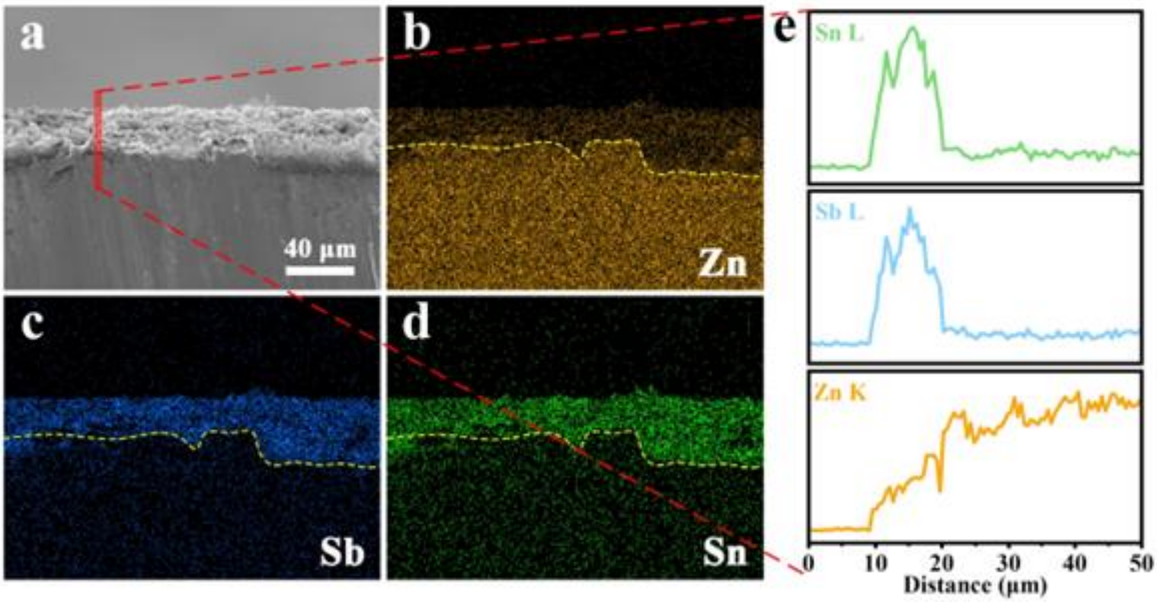

Figure S2. (a) Cross-sectional SEM, (b-d) corresponding EDS elemental mapping, and (e) linear scanning profiles of the SS@Zn electrode.

As shown in Figure S2a-d, the cross-sectional SEM image and corresponding elemental mapping images of the SS@Zn electrode show uniform elemental distributions of Zn, Sn, and Sb, which suggests the successful formation of SnSb alloy coatings on the surface of Zn foil. Moreover, the linear scanning profiles of SS@Zn electrode demonstrates that the thickness of SnSb alloy coatings is estimated to be ~10 μm.

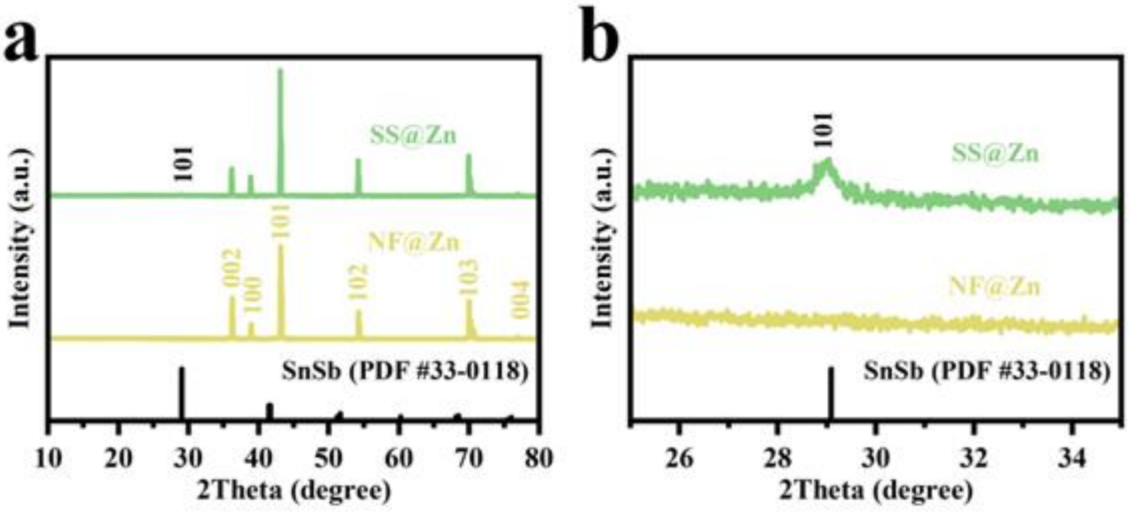

Figure S3. (a) XRD patterns and (b) amplified images of SS@Zn and NF@Zn electrodes.

Except for the Zn peaks from Zn substrate, the characteristic peaks of metallic SnSb (JCPDS No. 33-0118) can be clearly observed for the SS@Zn electrode (Figure S3), the intensity of which are however very weak due to poor crystallinity.

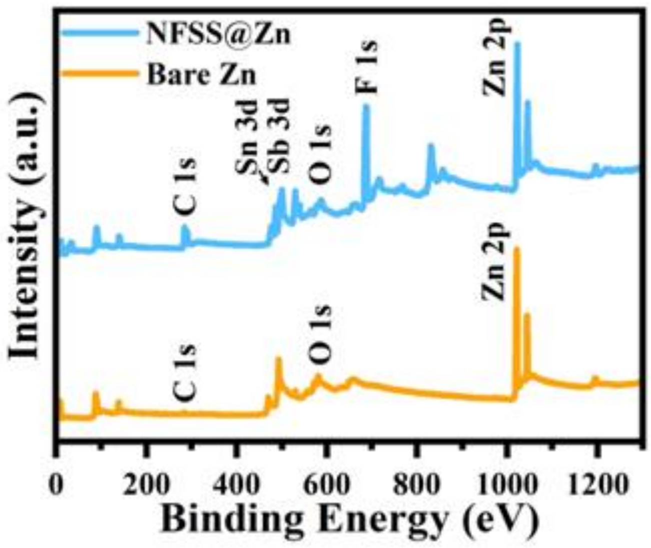

Figure S4. XPS survey spectra of the bare Zn and NFSS@Zn electrodes.

As shown in Figure S4, the XPS survey spectra of NFSS@Zn electrode give direct evidence for the existence of Zn, Sn, Sb, and F elements, in accordance with the above EDS mapping results.

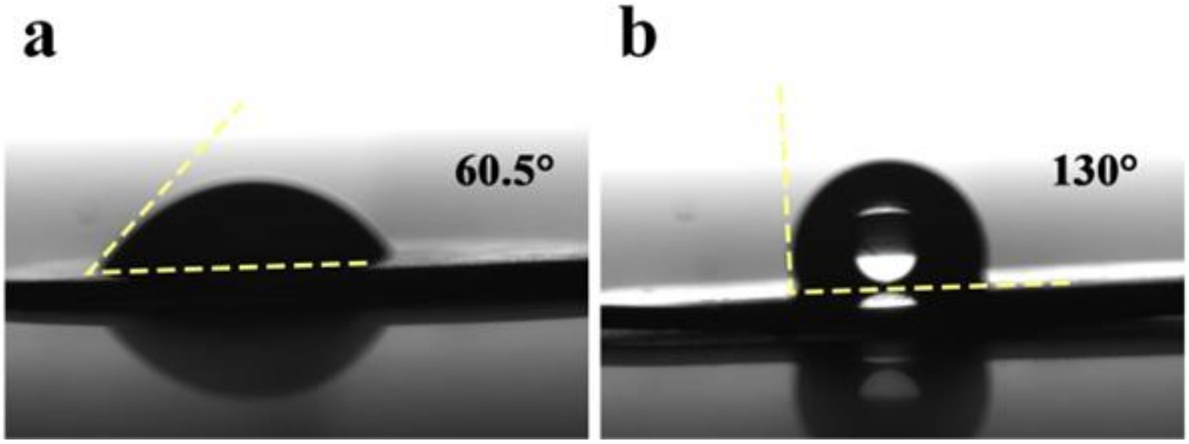

Figure S5. Contact angle measurement of (a) SS@Zn and (b) NF@Zn electrodes with 3 M $ZnSO_4$.

As shown in Figure S5, the contact angle of SS@Zn electrode is 60.5°, which is much smaller than that of NF@Zn electrode (130°). This result reveals that the SS protective layer can significantly improve hydrophilicity.

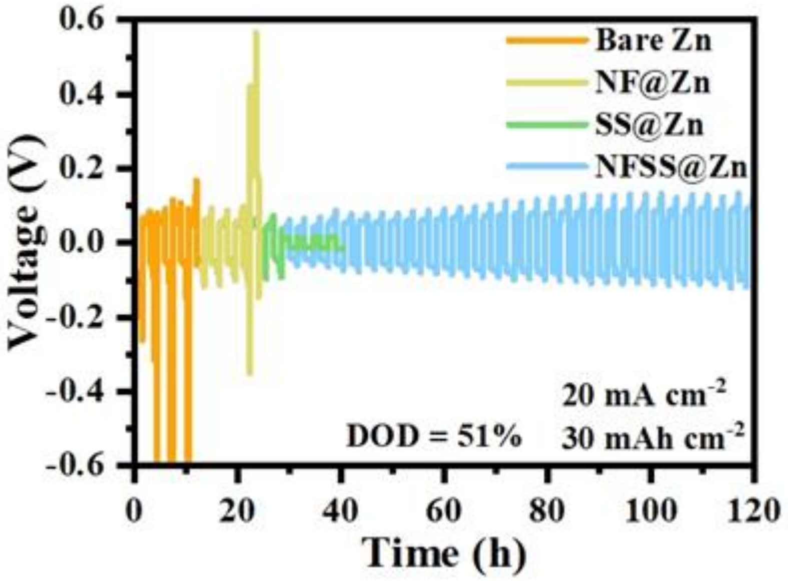

Figure S6. Cycling performance of symmetries cells with bare Zn, NF@Zn, SS@Zn, and NFSS@Zn electrodes at 20 mA cm^{-2} and 30 mAh cm^{-2}.

As shown in Figure S6, at a higher current density of 20 mA cm^{-2} and a cut-off capacity of 30 mA cm^{-2} (51% DOD), the NFSS@Zn electrode presents stable plating/stripping over 120 h, much better than 28 h for SS@Zn electrode, 22 h for NF@Zn electrode, and 3 h for bare Zn electrode. These tests obviously demonstrate that the NFSS layer can effectively inhibit the dendrite growth and promote stable Zn plating/striping, thereby extending the operational lifetime of the Zn anode.

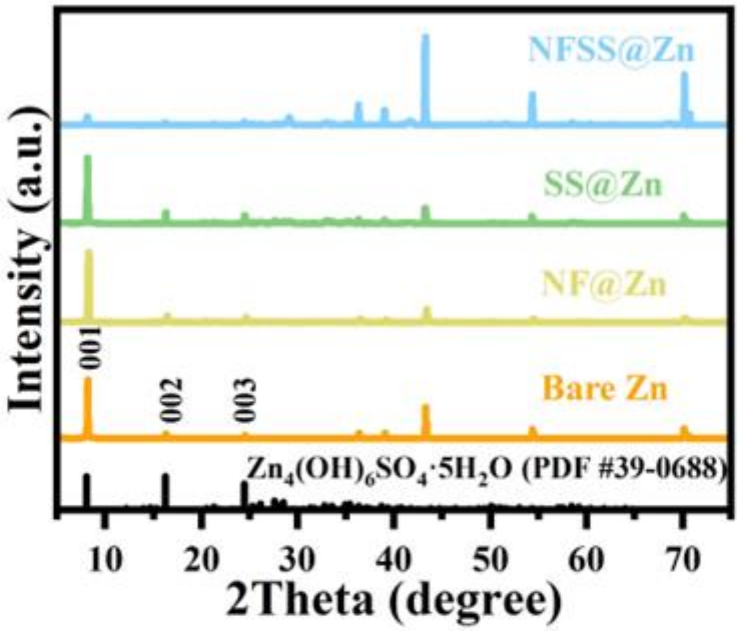

Figure S7. XRD patterns of bare Zn, NF@Zn, SS@Zn, and NFSS@Zn electrodes after 100 cycles at 6 mA cm^{-2} for 1 mAh cm^{-2}.

The bare Zn, NF@Zn, SS@Zn, and NFSS@Zn electrodes after 100 plating/stripping cycles were characterized using XRD, as shown in Figure S7. Obviously, three characteristic peaks at 8.06°, 16.22°, and 24.34°, which are indexed to the (001), (002) and (003) planes of $Zn_4SO_4(OH)_6{\cdot}5H_2O$ (JCPDS No. 39-0688), respectively, are detected in the cycled bare Zn, NF@Zn, and SS@Zn electrodes. In comparison, the diffraction peaks of $Zn_4SO_4(OH)_6{\cdot}5H_2O$ by-product are obviously reduced after the introduction of NFSS layer, again confirming the inhibition of side reactions by NFSS.

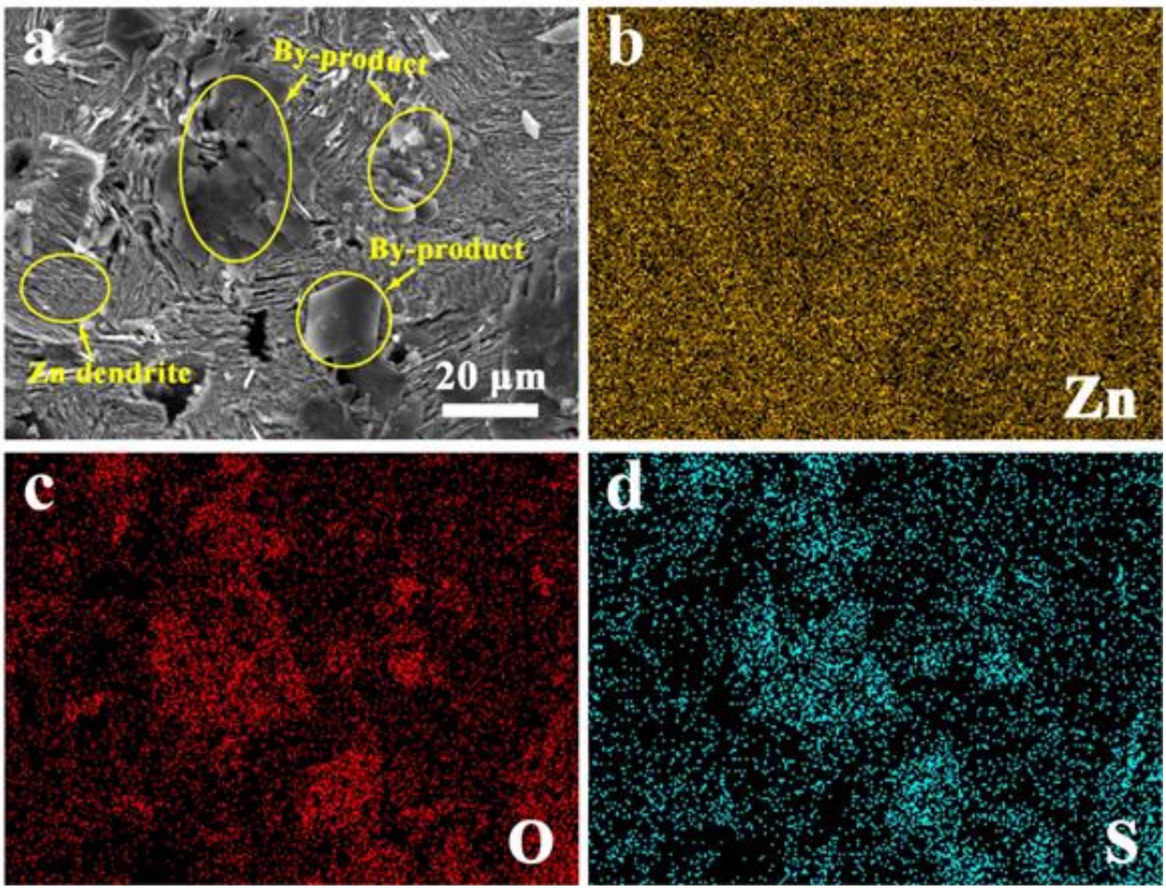

Figure S8. (a) SEM and (b-d) corresponding EDS maps of the cycled bare Zn electrode.

After 100 cycles, the surface of bare Zn is covered by numerous vertically growing flakes and irregular plate-like precipitates. The corresponding EDS maps of cycled bare Zn electrode show a large number of Zn, S, and O elements. The high Zn signal indicates the formation of $Zn_4SO_4(OH)_6{\cdot}5H_2O$ byproducts and Zn deposition.

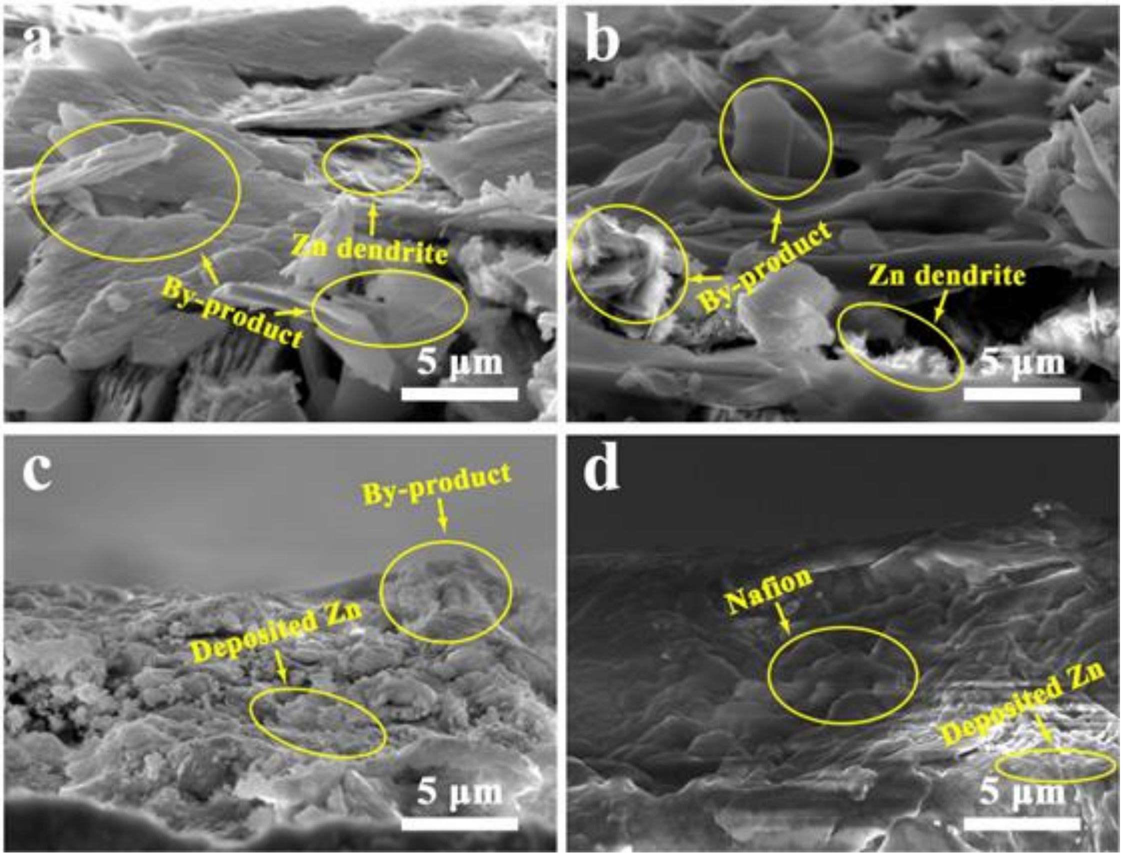

Figure S9. High-resolution cross-sectional SEM images of (a) bare Zn, (b) NF@Zn, (c) SS@Zn, and (d) NFSS@Zn electrodes after 100 cycles.

The surface morphologies of the cycled bare Zn, NF@Zn, SS@Zn, and NFSS@Zn electrodes were investigated by high-resolution cross-sectional SEM. As shown in Figure S9, a large pile of $Zn_4SO_4(OH)_6{\cdot}5H_2O$ mixed with vertical-growing Zn flakes is formed on cycled bare Zn electrode. After NF modification, the growth of Zn dendrites is effectively inhibited. However, there is still the presence of the flake-like $Zn_4SO_4(OH)_6{\cdot}5H_2O$. When a SS protective layer is introduced, the cycled SS@Zn electrode exhibits relatively uniform Zn deposition, but some granular-like protrusions are visible. In contrast, the cycled NFSS@Zn electrode shows complete protection against the growth of $Zn_4SO_4(OH)_6{\cdot}5H_2O$ and dendritic Zn flakes, suggesting highly efficient suppression of side reactions and dendrite growth.

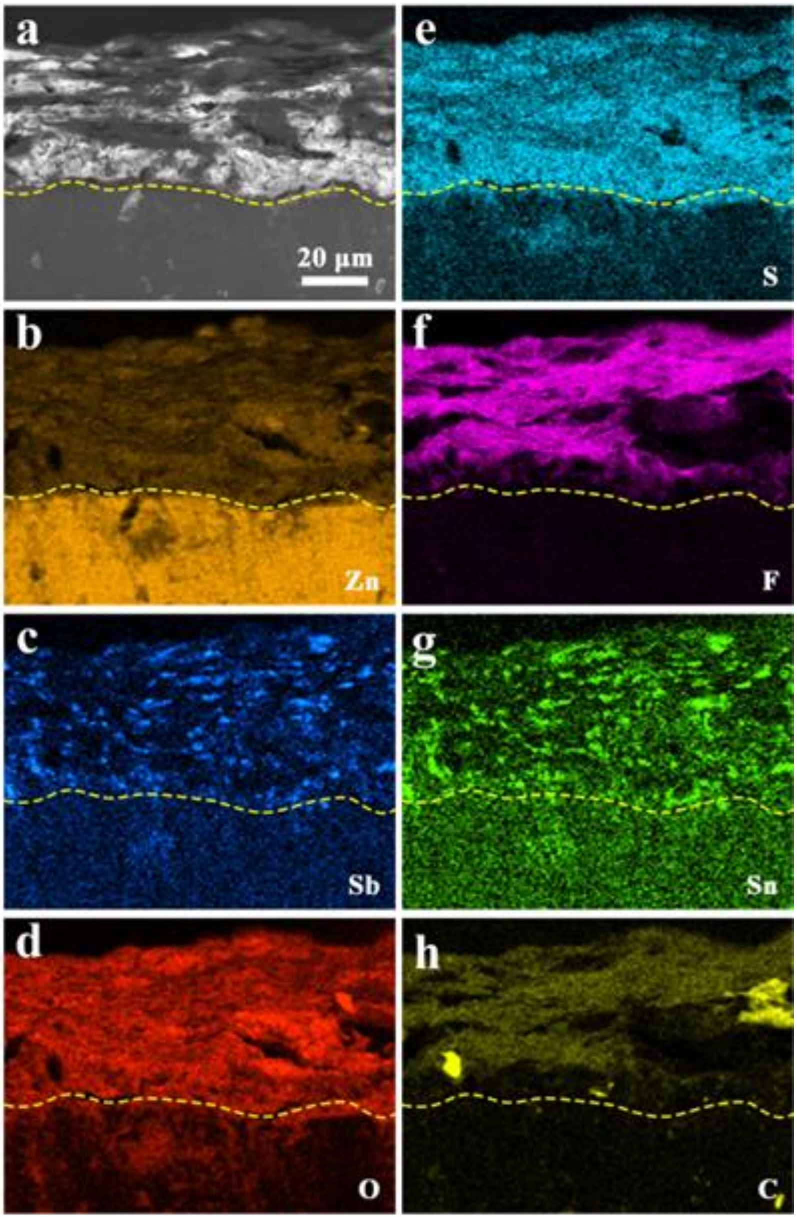

Figure S10. (a) SEM and (b-h) corresponding EDS maps of the NFSS@Zn electrode after 100 cycles.

As shown in Figure S10, after 100 cycles, the NFSS@Zn electrode still retains a flat and smooth surface without significant protrusions. EDS maps of the cycled NFSS@Zn electrode show uniform distribution of Zn, O, F, S, C, Sb, and Sn elements. It is worth noting that O, F, S, and

C arise from the Nafion, while Sn and Sb come from the SnSb alloy. Additionally, the EDS map of the Zn element appears below that of the F element, indicating that the Zn deposition occurs between the Nafion and SS layers.

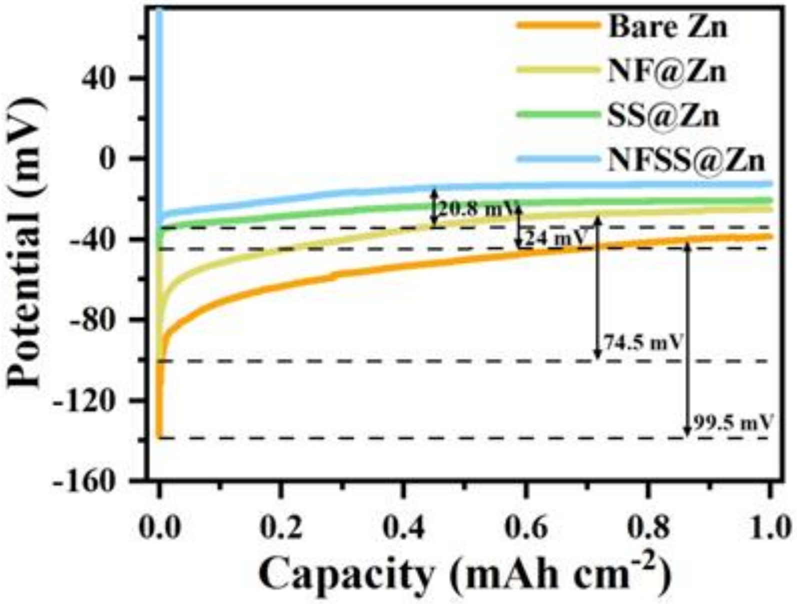

Figure S11. Galvanostatic voltage profiles of asymmetric cells using the bare Zn, NF@Zn, SS@Zn, and NFSS@Zn electrodes, respectively.

As shown in Figure S11, the NFSS@Zn electrode possesses a lower nucleation overpotential of 33.3 mV compared to bare Zn (138.3 mV), NF@Zn (99.8 mV), and SS@Zn (44.7 mV) electrodes, revealing the controllable and homogeneous Zn nucleation by the NFSS coating layer during Zn plating.

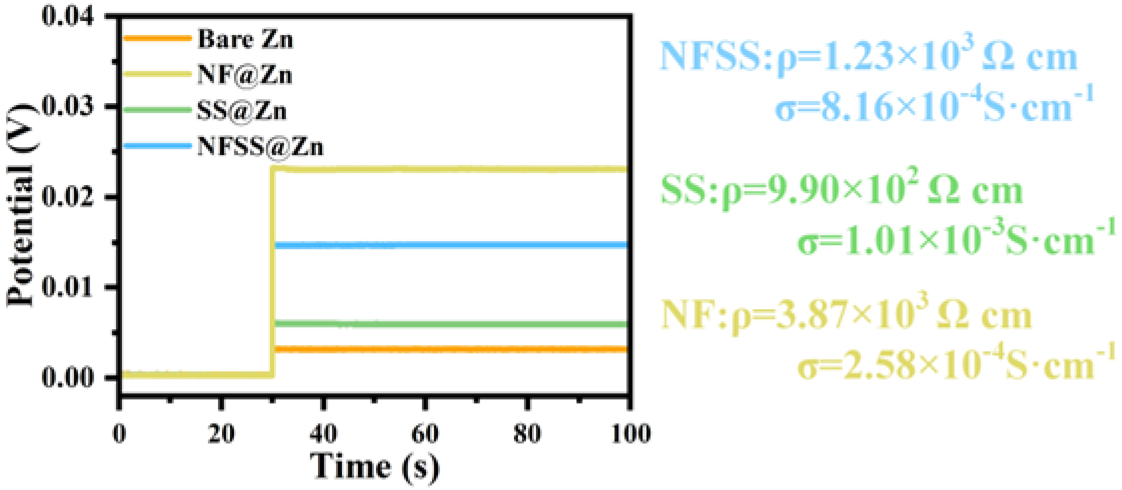

Figure S12. Measurements of the conductivity of Zn foil protected by the NF, SS, and NFSS layer, respectively. The orange curve reflects the voltage response to 3 mA current of bare Zn electrode that is sandwiched between two stainless steel spacers, and these green, purple, and blue curves are indexed to the SS@Zn, NF@Zn, and NFSS@Zn electrodes, respectively. The resistivity of the protection film was estimated according to the following equation: [S1]

$$\rho = \frac{R * S}{L} = \frac{U * S}{I * L}$$

L is the thickness of the protection film, *I* is the applied current, *R* is the resistance, *S* is the contact area between the stainless steel and the Zn foil (0.5 cm^{-2}), and *U* is the average voltage. The calculated values of the electronic resistivity for the NF, SS, and NFSS film are ~3.87×10^{3} Ω·cm ($\sigma_{electronic}$ = ~2.58×10^{-4} S cm^{-1}), ~9.90×10^{2} Ω·cm ($\sigma_{electronic}$ = ~1.01×10^{-3} S cm^{-1}), and ~1.23×10^{3} Ω·cm ($\sigma_{electronic}$ = ~8.16×10^{-4} S cm^{-1}), respectively.

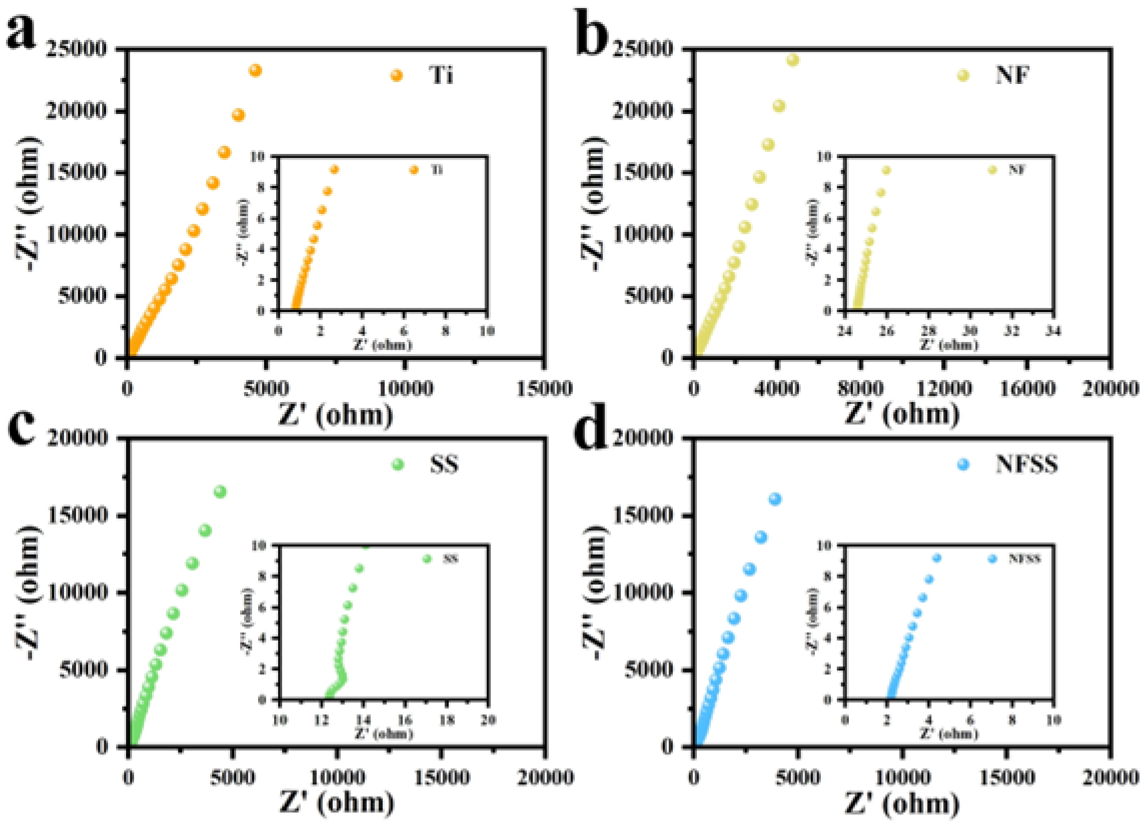

Figure S13. Nyquist plots measured at open circuit voltage (OCV) over the frequency range of 100 kHz to 0.1 Hz. (a) Ti symmetrical cells with glass fiber as separator. (b) NF, (c) SS, and (d) NFSS coated Ti symmetric cell with glass fiber as separator (inset: enlargement of indicated range). For the symmetric cells, the ionic conductivity of the film can be estimated based on the following equation: [S2]

$$\sigma = \frac{L}{R_b(modification\ layer)S}$$

where L is the thickness of film, S is the contact area (1 cm^{-2}), and $R_{b\ (glass\ fiber)}$ = ~0.8 ohm at 25 °C. For the decorated Ti symmetric cell, $R_{b\ (modification\ layer)} = R_b - R_{b\ (glass\ fiber)}$. Therefore, the ionic conductivities of NF, SS, and NFSS protective films can be estimated to be σ_{ionic} =4.20×10^{-5}, 8.62×10^{-5}, and 1.43×10^{-3} S cm^{-1}, respectively.

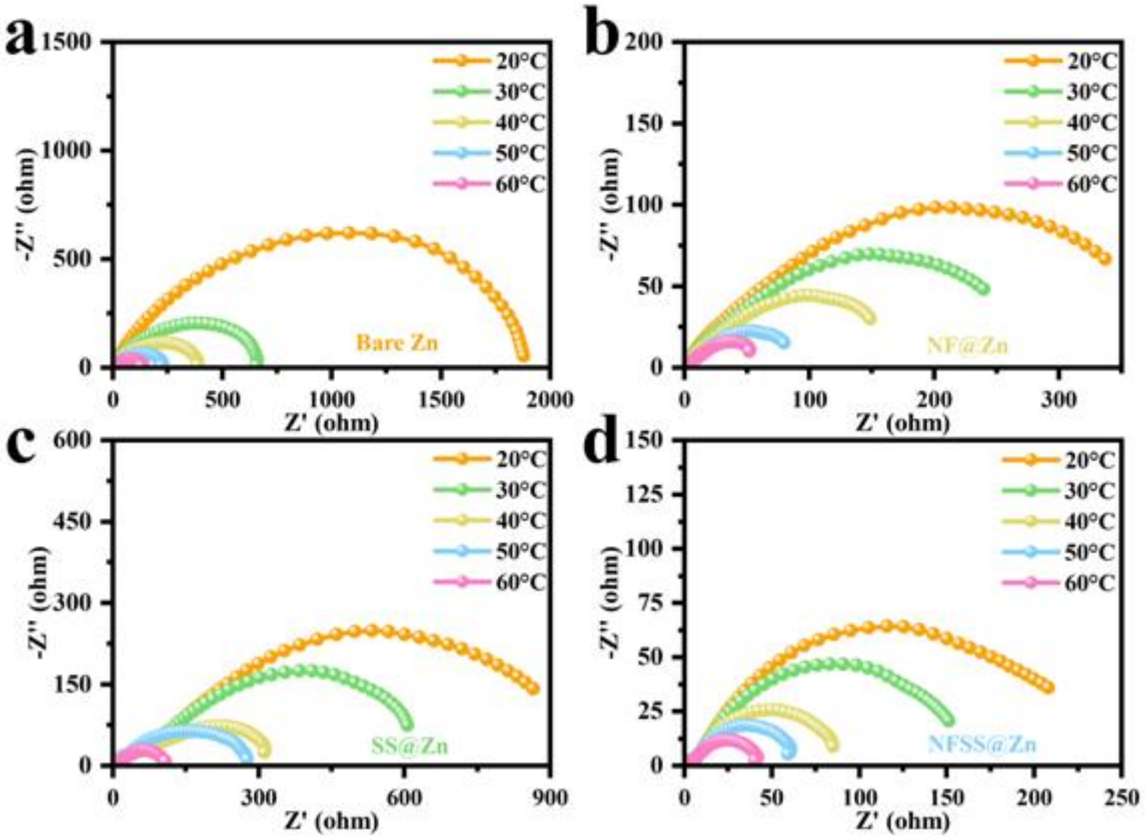

Figure S14. EIS spectra of bare Zn, NF@Zn, SS@Zn, and NFSS@Zn electrodes at various temperatures.

To calculate the activation energies of the transfer and desolvation of Zn^{2+} ions in the as-obtained samples, EIS measurements were conducted at different temperatures ranging from 20 to 60 °C. As shown in Figure S14, the R_{ct} values of NFSS@Zn display higher stability compared to those of bare Zn, NF@Zn, and SS@Zn electrodes as temperature increases. This result indicates that the NFSS layer can maintain interface stability and favor rapid charge transfer. Additionally, the observed reduction in activation energy (33.5 kJ mol^{-1}) for NFSS@Zn implies that the NFSS protective layer effectively enhances the desolvation of Zn^{2+}

ions, leading to efficient ion transfer processes and improved kinetics of Zn plating and stripping during cycling.

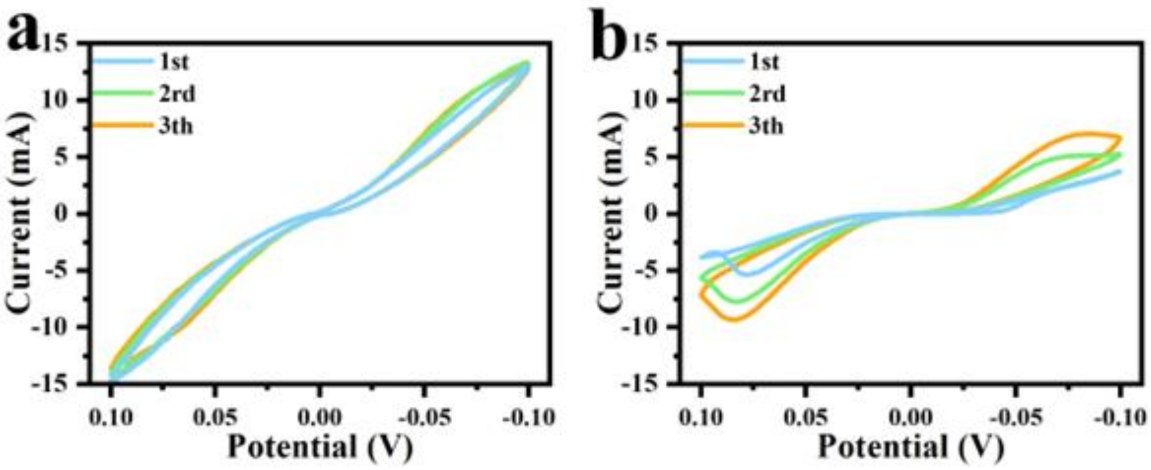

Figure S15. CV curves of the symmetric (a) NFSS@Zn and (b) bare Zn cells measured at 0.1 mV s^{-1}.

As shown in Figure S15a, CV curves of NFSS@Zn electrode in initial three cycles are well overlapped, indicating the good reversibility of Zn plating/stripping. However, the enclosed areas of CV curves of bare Zn electrode in the second and third cycles are much larger than that of the first cycle (Figure S15b).

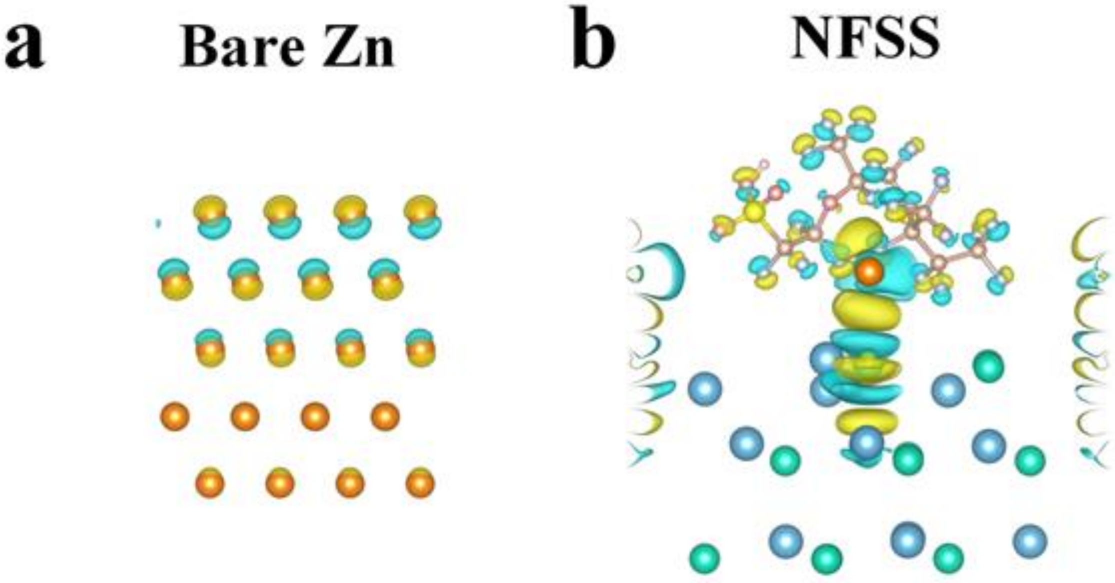

Figure S16. The charge density difference for Zn^{2+} absorbed on (a) bare Zn and (b) NFSS.

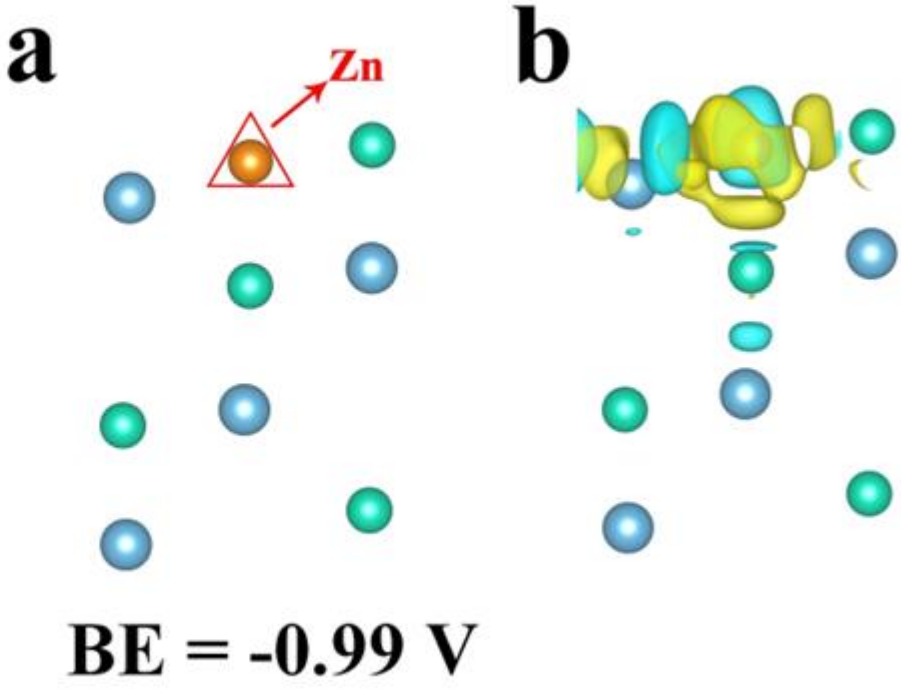

Figure S17. (a) Binding energy of Zn atom adsorbed on the top site of SnSb. (b) The charge density difference for Zn^{2+} absorbed on SnSb.

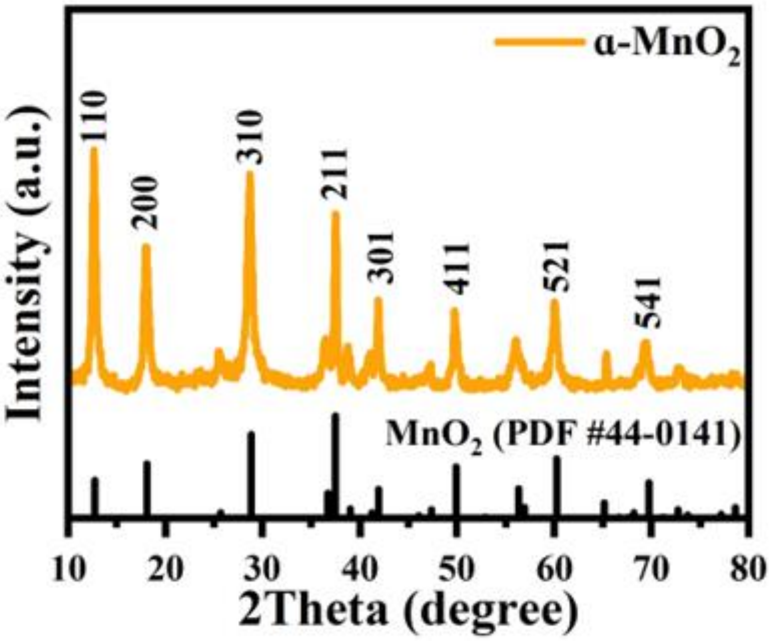

Figure S18. XRD pattern for MnO_2 nanowires.

As shown in Figure S18, all the diffraction peaks can be well indexed to the standard peaks of α-MnO_2 (JCPDS No. 44-0141) and no other characteristic peaks can be observed for impurities.

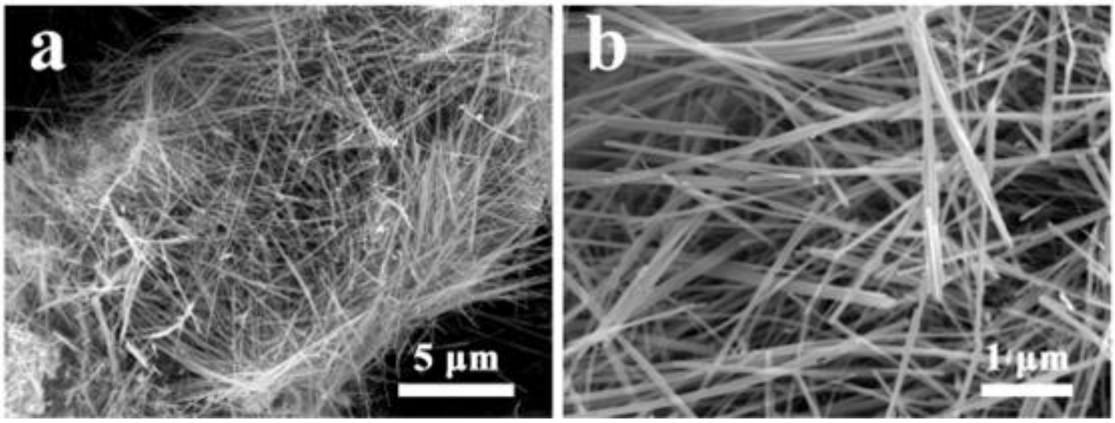

Figure S19. SEM images of MnO_2 nanowires at different magnifications.

As shown in Figure S19, the SEM images of MnO_2 show uniform morphology of nanowires with a length up to 10 μm.

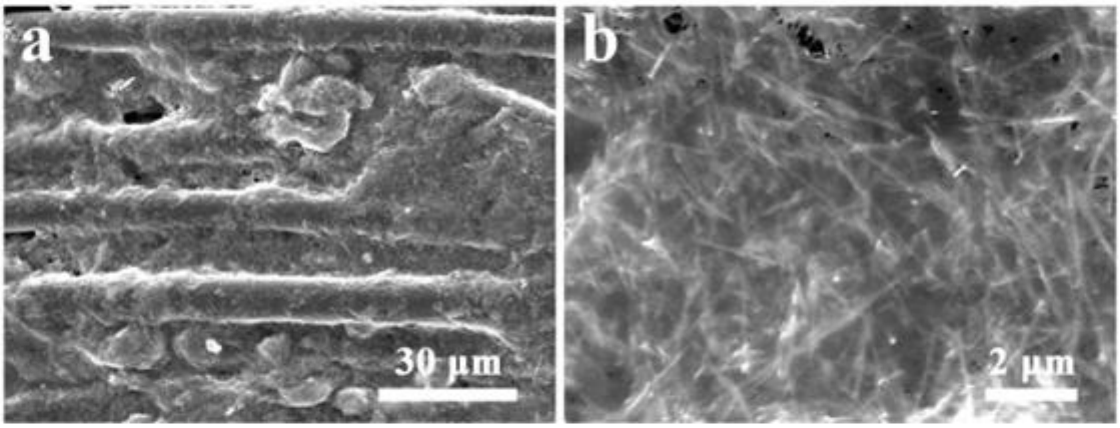

Figure S20. SEM images of pristine MnO_2 cathode at different magnifications.

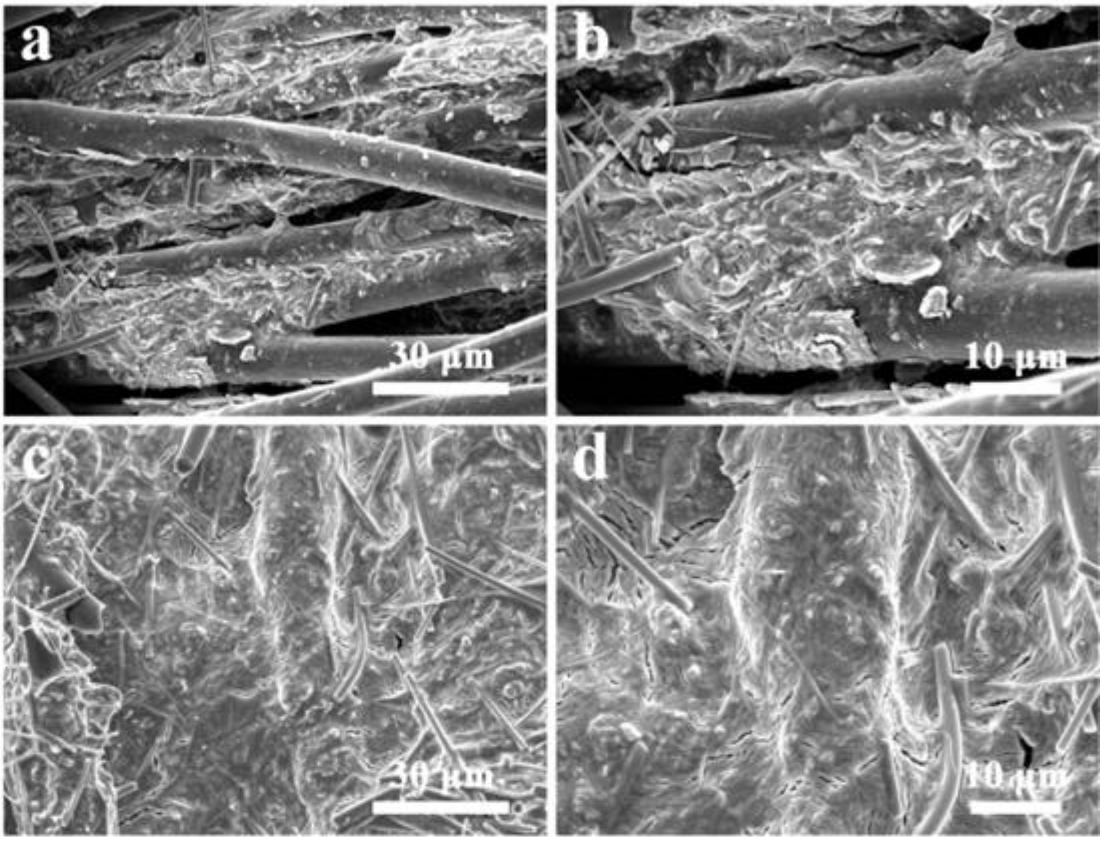

Figure S21. SEM images of (a, b) Zn||MnO_2 and (c, d) NFSS@Zn||MnO_2 full cells at different magnifications after cycling.

It is noted that the pH change in the electrolyte caused by the side reaction between Zn anode and electrolyte has a vital impact on the morphology of MnO_2 during the electrochemical reaction. As shown in Figure S21, bare Zn anode induces severe MnO_2 dissolution, leading to the loss of active material. However, the unique NFSS@Zn anode successfully suppresses the water-induced side reactions and hence MnO_2 nanowires dissolution during cycling, enabling a stable MnO_2 cathode for high-energy Zn-ion batteries.

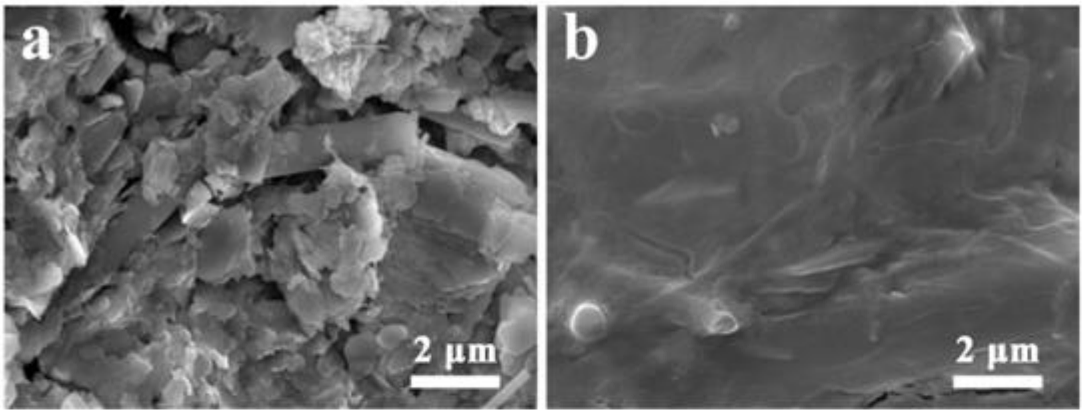

Figure S22. SEM images of (a) bare Zn and (b) NFSS@Zn electrode after cycling in full cells.

As shown in Figure S22, the bare Zn electrode surface after cycling in full cells become rough with flake-like by-products owing to the continuous side reaction of Zn with aqueous electrolyte. In contrast, the cycled NFSS@Zn electrode in full cells remain a dense and smooth morphology. Therefore, the NFSS protective layer holds great promise in suppressing the side reactions and regulating Zn deposition behavior.

REFERENCES

[S1]J. Hao, B. Li, X. Li, X. Zeng, S. Zhang, F. Yang, S. Liu, D. Li, C. Wu, Z. Guo, *Adv. Mater.* **2020**, 32, e2003021.

[S2]S. J. Wang, Z. Yang, B. T. Chen, H. Zhou, S. F. Wan, L. Z. Hu, M. Qiu, L. Qie, Y. Yu, *Energy Storage Mater.* **2022**, 47, 491.
